# From models to tools: clinical translation of machine learning studies in psychosis

**DOI:** 10.1038/s41537-020-0094-8

**Published:** 2020-02-14

**Authors:** Andrea Mechelli, Sandra Vieira

**Affiliations:** Department of Psychosis Studies, Institute of Psychiatry, Psychology & Neuroscience, King’s College London, London, UK

**Keywords:** Psychosis, Biomarkers

## Introduction

The past decade has seen a proliferation of neuroimaging-based machine learning studies in psychosis.^1^ Furthermore, within the span of ten years, small local studies with few dozen participants have evolved into large multi-centre studies with several hundreds of participants.^2–5^ In the midst of the search for accurate models, much attention has been given to methodological challenges including the impact of sample size,^6,7^ the limitations of traditional case–control designs,^8,9^ how to best deal with confounding variables^10^ and the effects of heterogeneity^11,12^ and inter-scanner variability,^13^ just to mention a few. Although there are still important methodological challenges to overcome, substantial progress is being made, and a solution to these challenges is now considered to be a matter of when rather than if.^14,15^ Wider discussions in the medical community about the ethical and legal implications of integrating machine learning models within diagnostic and prognostic assessment of patients are also underway.^16–20^ Taken collectively, the progress being made towards the development and validation of neuroimaging-based machine learning models is encouraging, as if the different pieces of a very complex puzzle were slowly coming together. Less discussed however, are the challenges related to the development and validation of machine learning-based clinical tools. Here the critical distinction is between “models”, which tend to be developed and validated using a limited number of well characterised datasets with the aim of maximising accuracy, sensitivity and specificity, and “tools”, which must be feasible, acceptable and safe, and provide information that will guide clinical decision-making in real-world settings. This is a timely discussion, as a new generation of multi-centre studies aiming to develop machine learning tools to manage patients with psychosis is emerging (e.g., PSYSCAN,^21^ PRONIA—www.pronia.eu).

Let’s imagine that we have developed a neuroimaging-based machine learning model with high levels of accuracy, sensitivity and specificity, after addressing the main methodological issues.^2^ Next we’d like to translate this machine learning model into an actual clinical tool to support the assessment of individual patients. What are our main challenges along this translation? In this opinion piece, we discuss seven critical aspects that require careful consideration when moving from a “model” to a “tool”. These include real-world validation, clinical utility, feasibility, acceptability, safety and finally, dissemination.

### Real-world validation

After validating our model using several independent datasets, collected using different scanners across multiple research sites, we might feel reassured about its performance in a real-world setting. Yet our optimism might be premature. This is because datasets collected for the purpose of research tend to include patients who meet stringent inclusion/exclusion criteria; unfortunately this highly selected group of patients differ from service users who do not take part in research (e.g., less severe, lower comorbidities, less medicated, and higher functioning).^22^ Therefore, when it comes to clinical validation, we need to consider not only the size but also the type of sample. In practice, the validation of a clinical tool should be done in a naturalist design, where all service users that may benefit from the tool are approached whilst having minimal exclusion criteria. It is likely that this will result in lower accuracies, sensitivities and specificities than the original validation using research datasets. The silver-lining here is that, if permitted, the more “naturalistic” data could be used to improve our tool. Learning from experience is, after all, one of the essential properties of machine learning.

### Clinical utility

Successful validation of our model using real-world data does not necessarily guarantee clinical utility. For a tool to be clinical useful, two conditions must be met. First, the tool must provide the treating clinicians with information which is not already available to them via conventional clinical assessment. Second, the tool must provide the treating clinicians with information that will influence a patient’s clinical management. Based on these conditions, there are at least four areas of potential clinical utility in the case of psychosis: (i) prediction of conversion to psychosis in individuals at clinical high risk (i.e., conversion vs. non-conversion); (ii) prediction of diagnostic outcome (i.e., affective vs. non-affective psychosis); (iii) prediction of response to conventional antipsychotic medication (responders vs. non-responders); and (iv) prediction of psychotic relapse amongst patients who have recovered from the acute phase of the illness (relapsers vs. non-relapsers). In each of these examples, it is not possible to predict clinical outcome based on the initial clinical presentation (condition 1), and the prediction has practical implications for clinical management (condition 2). A critical implication of this conceptualization of clinical utility is that very high levels of accuracy, sensitivity and specificity do not necessarily guarantee clinical utility. For example, a tool developed to distinguish between patients experiencing psychosis and healthy volunteers would not add to conventional clinical assessment, and as such would not be considered clinically useful even in the context of perfect accuracy, sensitivity and specificity. Conversely, accuracy, sensitivity, and specificity do not necessarily need to be very high for a tool to have clinical utility. For example, in circumstances where the clinical decision represents a “toss-up” and is subsequently adjusted via trial and error, even a modest improvement in prediction (e.g., from 50 to 70%) may be considered clinically useful. For completeness it should be mentioned that tools which do not meet both conditions, but have the potential of changing the patient’s perceptions and behaviours around their illness, could also have some clinical utility. For example, a tool predicting that someone has a very high chance of benefiting from a certain medication may make them more tolerant of side effects and less likely to discontinue treatment.

### Feasibility

Next we will need to establish the feasibility of implementing our tool in a real-world setting. In practice, this would require carrying out a prospective naturalistic investigation using both quantitative and qualitative methodologies. Feasibility from the perspective of healthcare providers could be assessed based on the proportion of clinical services, which have the capacity to collect the required neuroimaging data, and have access to the technical infrastructure to implement the tool. Here potential barriers include absence of scanning facilities and insufficient computational resources to process the images in a timely manner. Feasibility from the perspective of service users could be assessed based on the proportion of patients who meet the inclusion and exclusion criteria for our tool, and the proportion of acceptances, refusals and nonresponses. Here patients with acute psychosis might find the scanner environment too stressful, thereby limiting the feasibility of our tool in this phase of the illness. At present, the feasibility of implementing neuroimaging-based machine learning tools in clinical services for psychosis is unclear due to the paucity of empirical data.

### Acceptability

Not much is known about the acceptability of machine learning tools in health care services in what has been recognized as “blind spot” in AI research.^23^ Yet such knowledge is particularly important for mental health applications, where the use of AI may be perceived as more controversial.^24^ Negative attitudes towards AI-based clinical tools amongst clinicians and service users may pose a significant obstacle to translational implementation, and as such should be an integral part of any formal assessment of clinical viability. As part of our prospective naturalistic study, we would therefore need to measure acceptability from the perspectives of clinicians and service users, covering domains such as helpfulness, ease of use, attractiveness, safety, and privacy features. In addition, because a questionnaire might miss potential barriers, we might also want to carry out face-to-face qualitative interviews with both groups. These interviews will provide an opportunity to unearth and understand concerns about technical aspects (e.g., unreliable internet connection) as well as operational aspects (e.g., medical staff being uncomfortable or uncertain about how the output of our tool can be integrated into clinical assessment).

### Safety

A first source of risk in the translational implementation of our tool relates to the collection of brain scans. While MRI is generally regarded as a safe procedure, some service users may have conditions that may pose a risk (e.g., pregnancy). However, the screening for these conditions and other issues that may affect the quality of the image is a routine exercise in clinical settings, and as such should not be a significant barrier. On the other hand, experiencing stress and anxiety before and/or during the MRI is not uncommon,^25^ and services users with acute psychosis may find the procedure even more challenging.^26^ A second source of risk in the translational implementation of our tool relates to the possible misuse of its output by medical staff. Here the potential risk is that a clinician will misinterpret and/or misuse the information with detrimental consequences for the patient. As part of our prospective naturalistic study, we would therefore need to measure safety in terms of frequency of adverse events and inappropriate use of the tool by medical staff.

### Dissemination

Having established the clinical utility, feasibility, acceptability, and safety of our tool using real-world data, we are now ready to make it available to the wider clinical and research communities. This raises the question of how to best disseminate our healthcare innovation. This question requires careful consideration of our aims and values as healthcare innovators as well as the opportunities and limitations of the current market. On the one hand, we would like our tool to improve the way psychosis is diagnosed, monitored and treated, reducing the burden of the illness on patients, their carers and the wider society; in order to achieve this aim, it is imperative that our tool is developed for wide adoption and is made available to the widest possible cohort of patients. On the other hand, we need to ensure the long-term sustainability of our tool in light of its future running costs (e.g., technical updates, cloud-based hosting, and bug fixing); in order to achieve this aim a robust plan for market adoption, continuous development, and financial income generation over a sustained period will be required. We will therefore need to develop a dissemination and commercialization plan which combines these aims, covering aspects such as medical device regulations, unique value proposition, market size, and revenue streams, data governance and ethics, cost structure, partnerships, and key risks.^27^

## Conclusion

Neuroimaging-based machine learning studies of psychosis are generating a plethora of academic publications, many of which are reporting “promising” findings. The ultimate aim of our research, however, is to find ways of decreasing the burden of this illness on patients, their carers, and the wider society. In order to achieve this aim, we need to start translating these machine learning models into clinical tools. This translation is not straightforward, as it requires us to move away from the kind of metrics that are the cornerstone of academic publications (e.g., statistical significance of the accuracy) towards measures of feasibility, acceptability, safety and, of course, clinical utility. A further stumbling block is that the vast majority of published studies compared patients with psychosis against healthy volunteers using a cross-sectional design, and as such produced findings with little or no clinical utility. In contrast, to develop machine learning models capable of providing clinically useful information, we need access to longitudinal data (for example, whether a patient did or did not respond to a full cycle of conventional antipsychotic medication). In the near future, a number of ongoing large-scale studies using a longitudinal design are expected to come to completion (e.g., PSYSCAN,^21^ PRONIA—www.pronia.eu). It is hoped that the data resulting from these studies will provide our research community with opportunities to bridge the existing gap between models and tools.
